# Dexamethasone and Azathioprine Promote Cytoskeletal Changes and Affect Mesenchymal Stem Cell Migratory Behavior

**DOI:** 10.1371/journal.pone.0120538

**Published:** 2015-03-10

**Authors:** Natália Schneider, Fabiany da Costa Gonçalves, Fernanda Otesbelgue Pinto, Patrícia Luciana da Costa Lopez, Anelise Bergmann Araújo, Bianca Pfaffenseller, Eduardo Pandolfi Passos, Elizabeth Obino Cirne-Lima, Luíse Meurer, Marcelo Lazzaron Lamers, Ana Helena Paz

**Affiliations:** 1 Embryology and Cell Differentiation Laboratory, Experimental Research Center, Hospital de Clínicas de Porto Alegre, Ramiro Barcelos 2350, CEP 90035-903, Porto Alegre, RS, Brazil; 2 Graduate Program in Gastroenterology and Hepatology Sciences, Universidade Federal do Rio Grande do Sul, Ramiro Barcelos 2400, CEP 90035-903, Porto Alegre, RS, Brazil; 3 Experimental Research Center, Hospital de Clínicas de Porto Alegre, Ramiro Barcelos 2350, CEP 90035-903, Porto Alegre, RS, Brazil; 4 Morphological Sciences Department, Health Basic Sciences Institute, Universidade Federal do Rio Grande do Sul, Rua Sarmento Leite 500, CEP 90050-170, Porto Alegre, RS, Brazil; University of California Davis, UNITED STATES

## Abstract

Glucocorticoids and immunosuppressive drugs are commonly used to treat inflammatory disorders, such as inflammatory bowel disease (IBD), and despite a few improvements, the remission of IBD is still difficult to maintain. Due to their immunomodulatory properties, mesenchymal stem cells (MSCs) have emerged as regulators of the immune response, and their viability and activation of their migratory properties are essential for successful cell therapy. However, little is known about the effects of immunosuppressant drugs used in IBD treatment on MSC behavior. The aim of this study was to evaluate MSC viability, nuclear morphometry, cell polarity, F-actin and focal adhesion kinase (FAK) distribution, and cell migratory properties in the presence of the immunosuppressive drugs azathioprine (AZA) and dexamethasone (DEX). After an initial characterization, MSCs were treated with DEX (10 μM) or AZA (1 μM) for 24 hrs or 7 days. Neither drug had an effect on cell viability or nuclear morphometry. However, AZA treatment induced a more elongated cell shape, while DEX was associated with a more rounded cell shape (*P* < 0.05) with a higher presence of ventral actin stress fibers (*P* < 0.05) and a decrease in protrusion stability. After 7 days of treatment, AZA improved the cell spatial trajectory (ST) and increased the migration speed (24.35%, *P* < 0.05, *n* = 4), while DEX impaired ST and migration speed after 24 hrs and 7 days of treatment (-28.69% and -25.37%, respectively; *P* < 0.05, *n* = 4). In conclusion, our data suggest that these immunosuppressive drugs each affect MSC morphology and migratory capacity differently, possibly impacting the success of cell therapy.

## Introduction

Inflammatory bowel disease (IBD) is a family of chronic inflammatory disorders of the gastrointestinal tract, which includes Crohn’s Disease (CD) and ulcerative colitis (UC), and is characterized by the dysfunction of T cells and uncontrolled production of inflammatory cytokines [1]. Evidence indicates that IBD results from an interaction between genetic, environmental and microbial factors, resulting in an exaggerated and imbalanced mucosal immune response to the normal intestinal microflora. This inflammation is sustained by an alteration of the mucosal barrier and other immune system defects, which open possibilities for new treatments targeting immunomodulation and tissue repair [1–3].

IBD patients usually suffer from a poor quality of life and multiple adverse effects, and the disease remission often remains difficult to maintain. Despite improvements in current drug treatments, they are not entirely effective [1,4]. Furthermore, the incidence of IBD has increased in pediatric patients, who present a history of multiple intestinal resections and immune modulating treatments with or without biological agents. Their response in the long term is uncertain, which is one of the many reasons why there is a search for new therapies and why mesenchymal stem cells are being looked to as one of the best options to treat these inflammatory conditions [3].

Mesenchymal stem cells (MSCs) possess a fibroblast-like cell shape and are plastic-adherent; a panel of markers is used to help characterize these cells, along with differentiation into osteocytes, adipocytes and chondrocytes [5,6]. MSCs present great plasticity and multipotent capacity and have emerged as potent regulators of the immune response. These cells are known for having low immunogenicity, being able to escape recognition by T cells due to a low expression of HLA class I, and the lack of HLA class II and co-stimulatory molecules [7–9]. MSCs also secrete a variety of cytokines that suppress the local immune system, controlling inflammation and assisting in tissue repair [10–12]. These cells can be isolated from different organs and tissues, including bone marrow, muscle, adipose tissue, and feto-maternal organs. In addition, the use of postnatal placental tissue has shown several benefits as a source of MSCs [13–15]. When compared to other sources, placental-derived MSCs have been shown to possess a better proliferation rate [16] and superior engraftment capacity [17], to share some of the same markers encountered in embryonic stem cells [18] and to present increased immunosuppressive properties [19,20]. These cells also possess a great migration capacity both *in vitro* [21] and *in vivo* [22]. These results led to the successful administration of fetal-derived MSCs in a phase I study for the treatment of CD and UC [23].

To date, there have been competing theories over the mechanisms by which MSCs migrate to inflamed tissues. MSC homing is defined as the arrest of MSCs in the tissue vasculature followed by endothelial transmigration. Unlike the well-characterized adhesion cascade of leukocyte homing, there is currently an absence of a clear mechanism for MSC homing. The exact positioning of MCSs after infusion is unclear and makes it difficult to determine if cells have been arrested within the vessels or have gone through transendothelial migration [24]. Despite studies visualizing MSCs trapped in the lungs after intravenous infusion [25,26], several groups have found systemically administered MSCs reaching the target injured tissue, such as the brain [22,27], spinal cord [28], heart [29], colon [30] and kidney [31]. These data suggest that MSCs might have a homing capacity *in vivo* towards the injured site.

The use of cell therapies capable of local immunomodulation could be an alternative to improve current IBD outcomes, and different phase I-III clinical trials regarding IBD treatments using MSCs have resulted in promising and diverse outcomes [1,3,7,32–36]. MSC therapeutic approaches still rely on adequate cell homing towards the inflamed or injured site. Cell migration is a complex process that involves the break of cell polarity with the formation of a cell front and rear, which is characterized by the polymerization of actin and formation of nascent adhesions at the cell front, maturation of cell adhesions, contractility of cell body and detachment of adhesions at the cell rear [37–39]. Actin polymerization is involved in the formation of cell protrusions, and it depends on a fine regulation among several signaling and effector proteins [40]; the organization of the actin network is known to reflect different stages of cell migration [41,42]. The speed and the spatial trajectory of migrating cells are essential for the MSC homing process, and it relies on an efficient regulation of cell polarity and actin dynamics, which might be impaired by different physiological or pharmacological conditions [43,44].

IBD clinical treatments include administration of immunosuppressive drugs, such as azathioprine (AZA) and dexamethasone (DEX). Their immunosuppressive action targets the inhibition of purine nucleotide synthesis and the synthesis and metabolism of RNA or control the transcription of inflammatory genes, respectively [45,46]. Previous studies have analyzed immunosuppressive drug interactions with MSCs regarding cellular proliferation and functionality, including migratory chemotaxis capacity through transwell assay; however, while some studies conclude that these drugs might affect MSCs, there are others that show otherwise [47–51]. Considering cell therapy as a strong alternative for the treatment of IBD, it is imperative to study the interaction of MSCs and drugs commonly used in conventional treatment. To address this matter, we examined human chorion-derived MSCs for cell viability, nuclear morphometry, cell polarity, F-actin and FAK distribution, and cell migration in the presence of AZA or DEX at concentrations similar to those used in clinical treatments (1 μM and 10 μM, respectively) [1,48,52,53]. We observed early changes in cell polarity and cytoskeletal distribution (24 h) with DEX treatment. After 7 d, DEX impaired cell migration, while AZA was able to increase cellular speed; both results have strong relevance to MSC therapy outcome.

## Materials and Methods

### Ethics statement

Ethical approval was given by the Research Ethics Committee of Hospital de Clínicas de Porto Alegre (GPPG12–0082).

### Mesenchymal stem cells isolation and expansion

MSCs were isolated from human term chorionic membranes after written consent from the mothers. The tissue was separated (3 x 2.5 cm) and thoroughly washed with phosphate-buffered saline (PBS). It was minced and proteolytically digested with 1 mg/ml collagenase type I (Sigma, MO, USA) at 37°C for 1 h 45 min and centrifuged at 500 x *g* for 10 min at room temperature. Cells were plated in 6-well plates (TPP, Trasadingen, CH) using Dulbecco’s Modified Eagle’s Medium (DMEM; Gibco, CA, USA) low glucose supplemented with 20% fetal bovine serum (FBS; Gibco, CA, USA), 1% 100 units/ml penicillin and 100 mg/ml streptomycin (PS; Gibco, CA, USA) as standard medium. The media were replaced twice a week. For experiments, cells were used at passage 4–8, and the results of each experimental condition were compared among cells at the same passage.

### *In vitro* differentiation

In order to characterize MSCs in accordance with *The International Society for Cellular Therapy Statement* [5], two different experimental procedures were employed. Osteogenic differentiation was carried out using DMEM low glucose supplemented with 10% FBS, 1% PS, 0.1 μM dexamethasone, 10 mM β-glycerophosphate (Sigma-Aldrich, MO, USA) and 50 μM ascorbic acid 2-phosphate (Sigma-Aldrich, MO, USA), with 5000 cells/well in 24-well plates for 21 d. Differentiation was further confirmed by Alizarin Red (Sigma-Aldrich, MO, USA) staining [14].

Adipogenic differentiation was induced by culturing MSCs for 21 d in DMEM low glucose supplemented with 10% FBS, 1% PS, 1 μM dexamethasone, 0.5 mM isobutyl methylxanthine (Sigma-Aldrich, MO, USA), 10 μg/ml insulin and 200 μM indomethacin (Sigma-Aldrich, MO, USA). Adipogenic differentiation was confirmed by Oil Red (Sigma-Aldrich, MO, USA) staining [14].

### Flow cytometry analysis

In order to characterize the cell population according to surface molecular markers [5], immunophenotyping was performed. Cells were detached with trypsin-0.25% EDTA (Gibco, CA, USA) and washed by centrifugation (300 x *g*, 5 min) with PBS. Antibodies (CD73, CD90, CD105, CD45, CD34, CD14, CD19 and HLA-DR) were added to the cells (1x10^6^ cells) and incubated for 30 min at 4°C following the manufacturer’s protocol. Cells were then washed with PBS and analyzed using a BD FACSCalibur flow cytometer (Becton-Dickinson, NJ, USA) and Cellquest and PAINT-A-GAIT software. This analysis was also carried out after incubation of MSCs in the presence of AZA or DEX for 7d to ensure their stemness.

### Inflammatory bowel disease drugs

AZA and DEX (Sigma-Aldrich, MO, USA) were used at the relevant clinical concentrations of 1 μM and 10 μM, respectively. AZA was dissolved in dimethyl sulfoxide (DMSO; Sigma-Aldrich, MO, USA), and DEX was dissolved in DMEM low glucose. The AZA and DEX vehicle control groups were treated with complete standard medium supplemented with DMSO (0.5%) and DMEM low glucose, respectively. The range of concentrations of AZA and DEX used in the present study is comparable to those found in sera of patients with inflammatory conditions under these drugs treatments and is also commonly used for *in vitro* and *in vivo* studies [1,48,52,53].

### Cell viability by MTT assay

MSCs were seeded in a 24-well plate at 5000 cells/well and cultivated in the presence of drugs for 24 h or 7 d. After that, MSCs were incubated with standard medium containing 3-(4,5-dimethylthiazol-2-yl)-2,5-diphenyltetrazolium bromide (MTT; Sigma-Aldrich, MO, USA) (final concentration of 555.56 μg/ml) for 4 h at 37°C. At the end of the experiment, the medium was removed, and DMSO was added. The absorbance was read at 570 nm in a 96-well plate using a microplate reader (Biochrom Anthos Zenyth 200rt microplate reader, Cambridge, UK). Experiments were performed in quadruplicate from four independent assays [48,54].

### Nuclear morphometric analysis, actin cytoskeleton and immunofluorescence

Cells were plated (5000 cells/well) in a 24-well plate, which was followed by treatments with drugs for 24 h and 7 days. In sequence, cells were detached with trypsin-0.25% EDTA, washed and plated on fibronectin-coated glass coverslips (2 μg/ml, Sigma-Aldrich, MO, USA) and allowed to attach overnight (ON) in an incubator (37°C, 5% CO_2_). MSCs were washed with PBS, fixed with 4% paraformaldehyde and 4% sucrose for 15 min at room temperature (RT), and washed (PBS), and the membrane was permeabilized with PBS 0.3% Tween 20 (Sigma-Aldrich, MO, USA) for 10 min at RT. Cells were washed (PBS), blocked with normal goat serum (1:10, 1 h, RT), incubated with focal adhesion kinase (FAK; Cell Signaling) ON at 4°C, washed (PBS) and incubated with AlexaFluor 488 (Molecular Probes—Invitrogen, Oregon, USA) for 2 h at RT. After washing, actin staining was performed with rhodamine-phalloidin (1:100 in PBS, Molecular Probes—Invitrogen, Oregon, USA) for 1 h at 4°C, and coverslips were mounted using Prolong (Sigma-Aldrich, MO, USA) containing dye (DAPI) for nuclear staining and sealed with nail polish.

A nuclear morphometric analysis (NMA) was performed as described by Filippi-Chiela et al. (2012) [55] on an Axio Observer Z1 microscope (Zeiss, Göttingen, Germany) with a charge coupled device camera (Axiocam mrn, Zeiss, Göttingen, Germany) using a 10x objective (Ecplan-Neofluar 10x/0.3 aperture, Zeiss, Göttingen, Germany) and AxioVision Software (Zeiss, Göttingen, Germany). Briefly, DAPI-stained cells were excited with a mercury lamp and an excitation filter (EX G 365, EM BP445/50). Images were taken from a total of 100–300 nuclei, obtained from random fields, from 4 independent experiments. Images were analyzed using Image J Software, for the acquisition of the nuclear area and the parameters of nuclear irregularity (roundness, aspect, radius ratio and area/box, which are grouped in an index, named the nuclear irregularity index (NII)). The plot of nuclear area per NII permits the separation of different nuclear populations and the inference of different cell death and growth inhibition mechanisms such as apoptosis (small and regular nuclei—SR nuclei), senescence (large and regular nuclei—LR nuclei) and irregular nuclei (I nuclei) [55,56].

Actin staining and FAK images were obtained with a confocal microscope (Leica TCS SP5 II, Wetzlar, Germany) using a 40x objective (HCX PL APO 40x/1.30 oil immersion). Rhodamine was excited using the 514 nm laser line of an Argon laser; DAPI was excited with a 405 nm laser line of a 405 Diode laser; and Alexa488 was excited with 488 nm laser line of an Argon laser (Melles Griot, Albuquerque, NM). Fluorescence images were acquired using Leica LASAF Software (Leica, Wetzlar, Germany). In each experiment (*n* = 4), 10 images per condition were collected (total of 40 images), and the most representative samples of each set of experiments were selected.

In order to quantify stress fiber density, 20 images per condition were randomly selected, and the analysis was carried out using Image J software as previously described [57]. After the elimination of background fluorescence using the *threshold* tool, a perpendicular line was drawn at the protrusion extension, and the average pixel intensities were measured using the *plot profile* function. The stress fiber density ratio was calculated as the pixel values greater than the threshold limit divided by the total line distance. Additionally, the total number of dorsal and ventral stress fibers per cell was quantified, as described by Kovac et al. (2013) [58].

### Cell polarity

To assess cell polarity, the polarity index was calculated as the length of the major migration axis (parallel to the membrane protrusion) divided by the length of the perpendicular axis that intersects the center of the cell nucleus (S1 Fig.) [44]. A total of 80–100 live cells were analyzed in each condition from 4 independent experiments.

### Migration assay

Cells were plated in a 6-well plate (6x10^4^ cells/well) and then treated with drugs or vehicle media for 24 h or 7 d. After the treatments, cells were detached with trypsin-0.25% EDTA, washed and plated on fibronectin-coated glass-bottomed dishes (2 μg/ml) in DMEM low glucose 10% FBS with or without drugs for 1 h, and the cells were then maintained in an incubator at 37°C. For analysis of the migration properties, phase microscopy time-lapse images were captured for a period of 20 h at 10 min intervals (migration speed and spatial trajectory (ST)) or 50 min at 10 s intervals (protrusion activity) (Ecplan-Neofluar 10x/0.3 aperture objective) with a charge coupled device camera (Axiocam mrn, Zeiss, Göttingen, Germany) attached to an inverted microscope (Axio Observer Z1, Zeiss, Göttingen, Germany) using AxioVision Software (Zeiss, Göttingen, Germany). The values for the assessment of migration speed and ST were obtained using Image J (*National Institute of Health*, MA, USA) software, and the data were processed as previously described [44]. For ST analysis, a polar plot graph was constructed, which represents the spatial trajectory developed by each migratory cell, where the X and Y coordinates of each cell trajectory were normalized to start at a virtual (X = 0 and Y = 0) position. A total of 80–100 cells were analyzed from 4 independent experiments for each condition.

### Statistics

Experimental data are presented as the mean ± SEM, unless indicated otherwise. Statistical analyses were performed using GEE (Generalized Estimated Equations) for viability, Kruskal-Wallis post-hoc Dunn for Polarity Index analysis, ANOVA post-hoc Student-Newman-Keuls (SNK) for actin quantification and ANOVA post-hoc Tukey HSD for migration speed in PASW Statistics (SPSS—version 18.0). ANOVA post-hoc Student-Newman-Keuls (SNK) for multiple comparisons was used for nuclear morphometric analysis in GraphPad INSTAT (GraphPad Software, San Diego, California, USA). From these analyses, *P*-values less than 0.05 were considered significantly different.

## Results

### Isolation and characterization of chorion-derived MSCs

**Fig 1:**
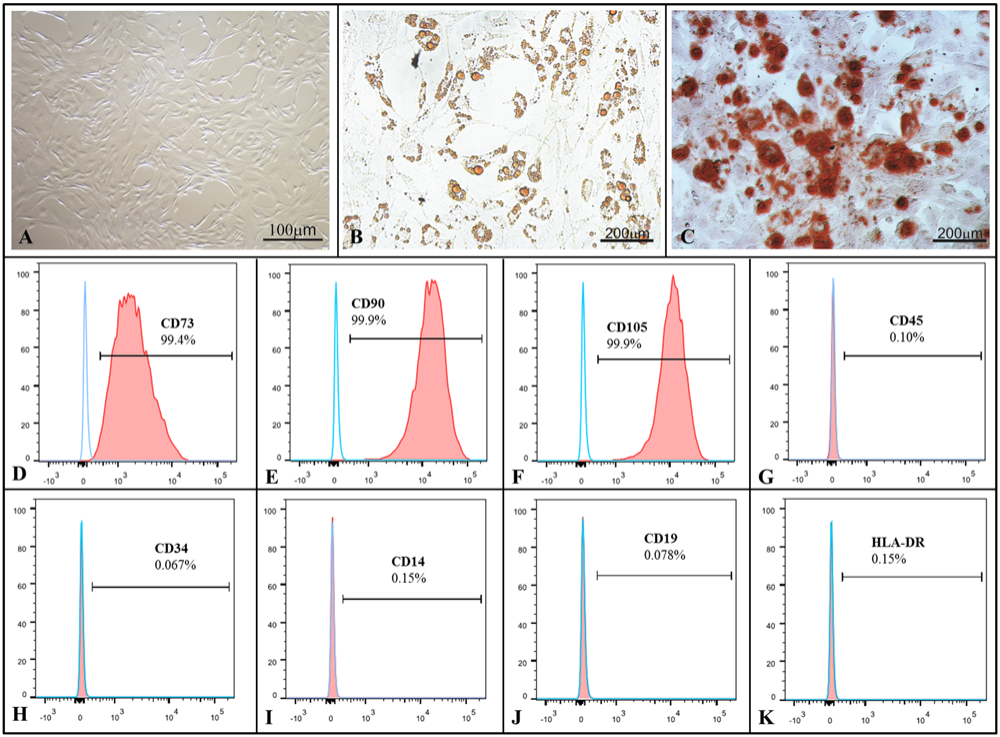
MSC characterization. Human chorionic-derived stem cells exhibit a fibroblast-like cell shape **(A)** and showed successful adipogenic **(B)** and osteogenic **(C)** differentiation potential, presenting a positive signal for **(D-F)** CD73, CD90 and CD105 and no signal for **(G-K)** CD45, CD34, CD14, CD19 and HLA-DR markers (*n* = 4).

MSCs from human chorion membrane were isolated and cultured, presenting a fibroblast-like cell shape as expected (Fig. 1A). To investigate the differential potential of MSCs, adipogenic and osteogenic differentiation was induced *in vitro*. A clear potential for adipogenic differentiation was detected by oil red staining of lipid vacuoles (Fig. 1B), and osteogenic differentiation was confirmed by alizarin red staining of calcium deposits (Fig. 1C). Cells expressed the mesenchymal markers CD73 (99.4%), CD90 (99.9%) and CD105 (99.9%), but they lacked CD45 (0.10%), CD34 (0.067%), CD14 (0.15%), CD19 (0.078%) and HLA-DR (0.15%) (Fig. 1D-K).

### MSCs maintain their stemness in the presence of immunosuppressive drugs

**Fig 2:**
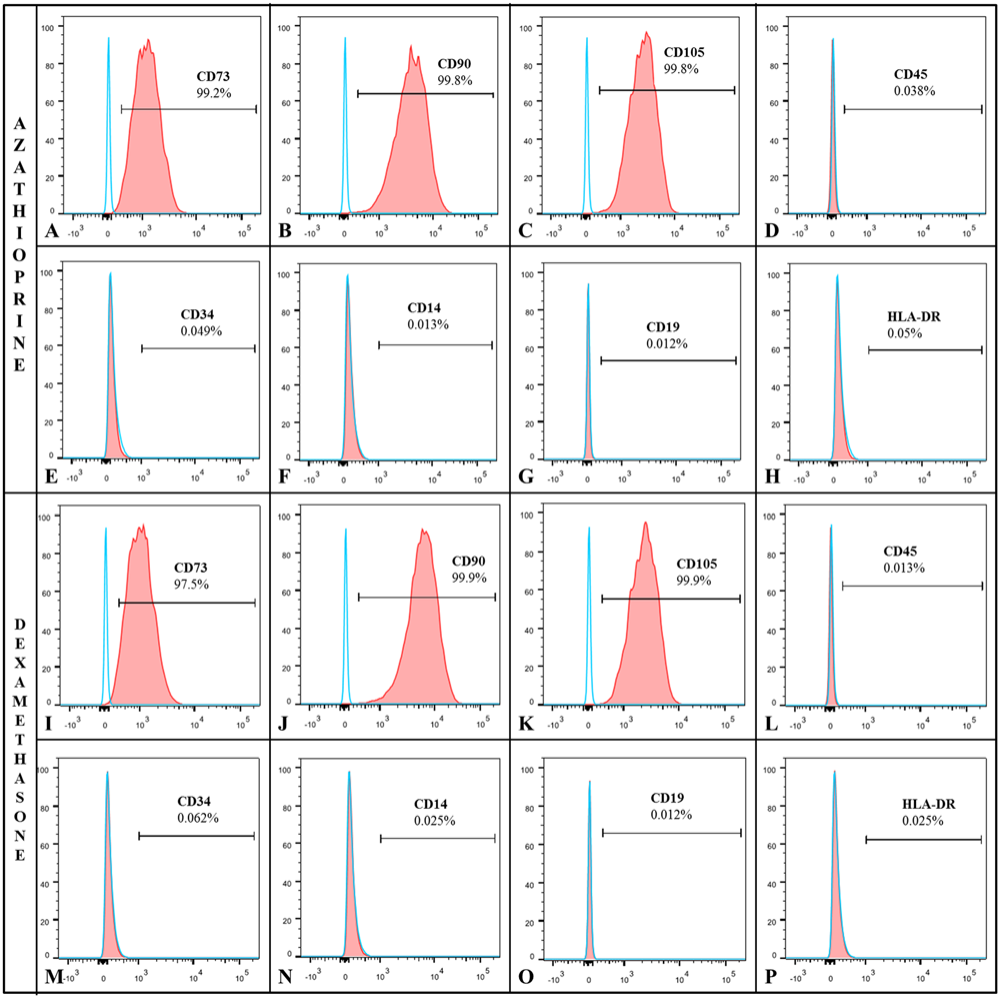
MSC immunophenotyping under DEX or AZA treatments. MSCs were cultured for 7 d under AZA or DEX treatments, and immunophenotyping analysis was performed in order to ensure that the cells still maintained their stemness. MSCs under AZA **(A-H)** or DEX **(I-Q)** treatments presented a positive signal for CD73, CD90 and CD105 but no signal for CD45, CD34, CD14, CD19 and HLA-DR markers (*n* = 4).

MSC immunophenotyping was preserved in the presence of AZA and DEX after 7 d of cultivation. Cells in the presence of AZA expressed the mesenchymal markers CD73 (99.2%), CD90 (99.8%) and CD105 (99.8%), but not CD45 (0.038%), CD34 (0.049%), CD14 (0.013%), CD19 (0.012%) and HLA-DR (0.05%) (Fig. 2A-H). Additionally, cells in the presence of DEX expressed the mesenchymal markers CD73 (97.5%), CD90 (99.9%) and CD105 (99.9%), but not CD45 (0.013%), CD34 (0.062%), CD14 (0.025%), CD19 (0.012%) and HLA-DR (0.025%) (Fig. 2I-P).

### Cell viability and nuclear morphometry are not affected by IBD drugs

**Fig 3:**
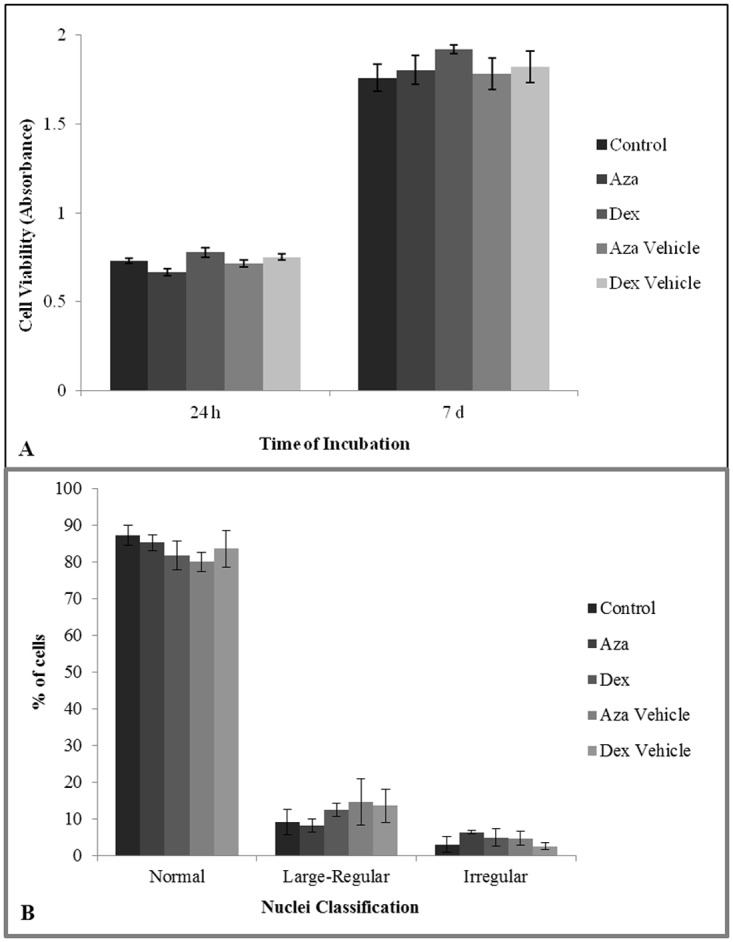
MSC viability and nuclear morphometry. MSCs were cultured for 24 h or 7 d under AZA 1 μM or DEX 10 μM treatments and assessed for cell viability through MTT assay **(A)** and nuclei irregularity index assessment **(B)**. All groups and experiments were compared, resulting in no difference between control and treatments (*n* = 4, *P* > 0.05). The time point in 2B is 7 days of treatment, and the 24 h time point is not shown. The MTT assay was analyzed using the GEE test, and nuclear morphometry was analyzed using ANOVA post-hoc SNK.

Changes in cell viability could result in a diminished number of cells and a low effectiveness of cell therapy. In order to verify the effects of drugs on MSC viability, cells were incubated with immunosuppressive drugs for 24 h and 7 d, and mitochondrial dehydrogenase activity was measured in the living cells by MTT assay [54]. The results showed no difference at the enzyme level under DEX and AZA treatments (Fig. 3A, *P* > 0.05, *n* = 4). Therefore, nuclear morphometry can indicate several cell fates such as early apoptosis and senescence, which may compromise MSC quality for therapeutic purposes. In order to analyze the nuclear morphometry, NMA was carried out, and no difference was observed in nuclear morphometry at all different drug treatments and time points (Fig. 3B, *P* > 0.05, *n* = 4). Altogether, our results from the viability analysis and NMA show that DEX and AZA treatments are not toxic for MSCs.

### IBD drugs change the polarity profile of the cells

**Fig 4:**
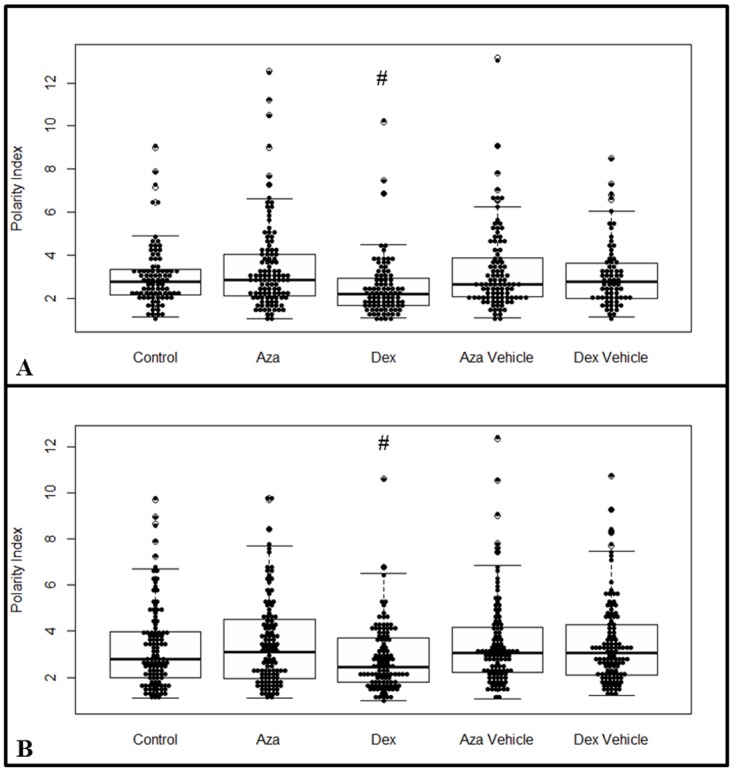
Cell Polarity Index. MSCs were cultured for 24 h **(A)** or 7 d **(B)** with or without AZA 1 μM or DEX 10 μM, and their polarity index was measured according to S1 Fig. PI values were obtained from 80–100 cells (*n* = 4), and the results showed that DEX treatment induced a more rounded cell shape (^#^ *P* < 0.05), while AZA showed a tendency to induce more elongated cells when compared to the control group. Graphs present the median value between the lower and upper quartiles, while showing the variability outside this range. The polarity index assay was analyzed using Kruskal-Wallis post-hoc Dunn.

Cell polarity results from the internal organization of the cell and is a key step for the induction of cell motility, where a more elongated morphology is usually associated with a better mesenchymal cell migratory performance [59,60]. The drugs’ effects on cell polarity at each time point (24 h and 7 d) were assessed through a polarity index, where the values closer to 1.0 represent a rounded cell shape and values higher than 3.0 represent elongated cells (Fig. 4A and 4B, respectively; S1 Fig.). AZA and DEX vehicles had no effect on the polarity index when compared to the control group. AZA tended to induce elongated shaped cells (2.8 (2.1; 4.1) vs. 2.7 (2.1; 3.3) in 24 h, 3.08 (1.9; 4.5) vs. 2.7 (1.9; 3.9) in 7 d; AZA vs. Control, respectively), while DEX treatment resulted in a higher presence of rounded shape cells (2.1 (1.6; 2.9) vs. 2.7 (2.1; 3.3) in 24 h, 2.4 (1.7; 3.7) vs. 2.7 (2.1; 3.3) in 7 d; DEX vs. Control, respectively, *P* < 0.05). These data suggest that DEX could impair the migratory activity of MSCs.

### IBD drugs alters MSC actin organization

**Fig 5:**
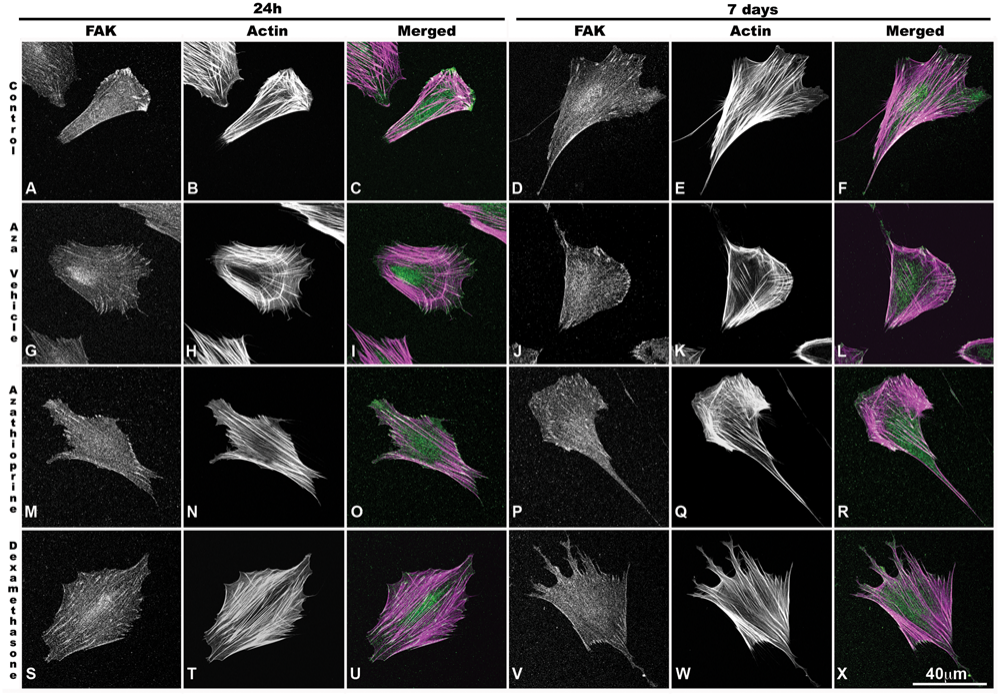
Actin cytoskeleton and FAK distribution. MSCs were cultured for 24 h or 7 d with or without drugs, plated on fibronectin-coated dishes overnight, fixed and stained for focal adhesion kinase (FAK) and actin analysis. FAK (green) and actin (magenta) co-localization (white) is demonstrated on merged images. Control cells showed small adhesions at the cell border **(A and D)** and actin stress fibers **(B and E)** that showed no variation in the presence of AZA vehicle for 24 h **(G-I)** or 7 days **(J-L)**. Cells treated with AZA (1 μM) presented large adhesions at the cell rear and smaller adhesions at the cell cortex **(M and P),** the presence of ventral stress fibers **(N)** and a tendency of increased presence of dorsal stress fibers **(Q)**. After 24 h or 7 days of DEX treatment, there was an increase in adhesion size throughout the cell body **(S and V)** and ventral stress fibers **(T, W)**, which was followed by an increase in instable membrane projections at 7 days **(X)**. Bar = 40 μm; representative images of *n* = 40 cells per condition.

**Fig 6:**
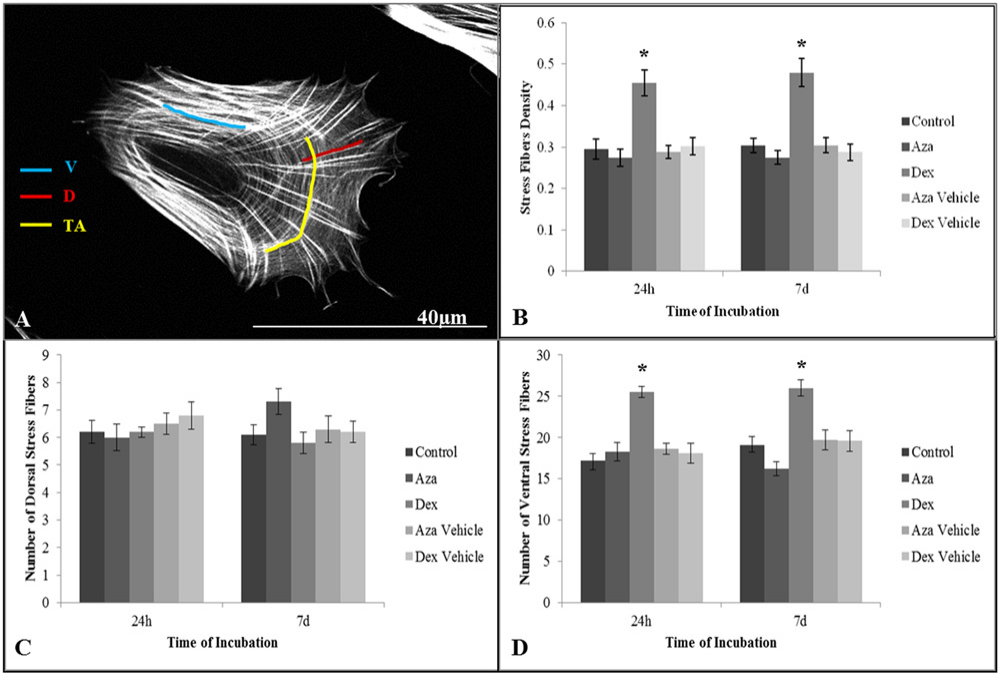
Actin analysis. MSCs were cultured for 24 h or 7 d with or without drugs and stained with rhodamine-phalloidin in order quantify actin density and dorsal or ventral stress fibers **(A)** through Image J. MSCs treated with DEX presented an increase in actin density **(B)**, consistent with a higher presence of ventral actin stress fibers **(D)**. None of the drugs seemed to alter significantly the presence of dorsal stress fibers **(C)**, while AZA did not show any difference in actin density **(B)** and ventral stress fibers **(D)**. *P < 0.05, *n* = 20. D = Dorsal stress fibers; V = Ventral stress fibers; TA = Transversal arcs of stress fibers. Actin quantification was analyzed through ANOVA post-hoc SNK. Bar = 40 μm.

Actin filaments are responsible for changes to the cell shape in response to stimuli, coordinating cellular protrusions and locomotion [38]. We observed morphological changes when drugs were added to the culture media; DEX induced a higher presence of stress fibers, while AZA preserved some of the lamellipodia (Fig. 5A-X; S2 Fig.). In mesenchymal cells, filamentous actin is observed as dorsal, transverse arcs or ventral stress fibers (Fig. 6A), where dorsal fibers are associated with a better migratory output, while ventral fibers reflect a more contractile and slower migratory phenotype [42,58]. We observed that the AZA 7 d treatment tended to induce an increase in the presence of dorsal stress fibers (Fig. 5Q), while the DEX 24 h and 7 d treatments were associated with a higher actin fiber density (54.7% and 58.2%, respectively, in comparison to control group; Fig. 6B, *P* < 0.05), with a predominance of ventral stress fibers (48.2% and 36.1%, respectively, in comparison to control group; Fig. 6D, *P* < 0.05). The analysis of the cell adhesion marker, FAK, correlated with these findings, with DEX inducing the presence of larger adhesions throughout the cell body (Fig. 5S and 5V), while AZA and control cells showed the presence of large adhesions at the cell rear and smaller adhesions at the cell cortex (Fig. 5A, 5D, 5M and 5P). To confirm these changes in the actin network organization and adhesion properties, we performed time-lapse movies in order to analyze the protrusion dynamics. Control, vehicle-treated and AZA 24 h and 7 d cells showed stable protrusions (Fig. 5A-R; S1 and S2 Movies); however, after 24 h of DEX treatment, MSCs presented increased membrane activity that was followed by the presence of unstable protrusions after 7 days of treatment (Fig. 5S-X; S3 Movie). These data suggest that immunosuppressive drugs may have different effects on the actin dynamics of MSCs, with possible effects on its migratory activity.

### Dexamethasone impairs MSC migration

**Fig 7:**
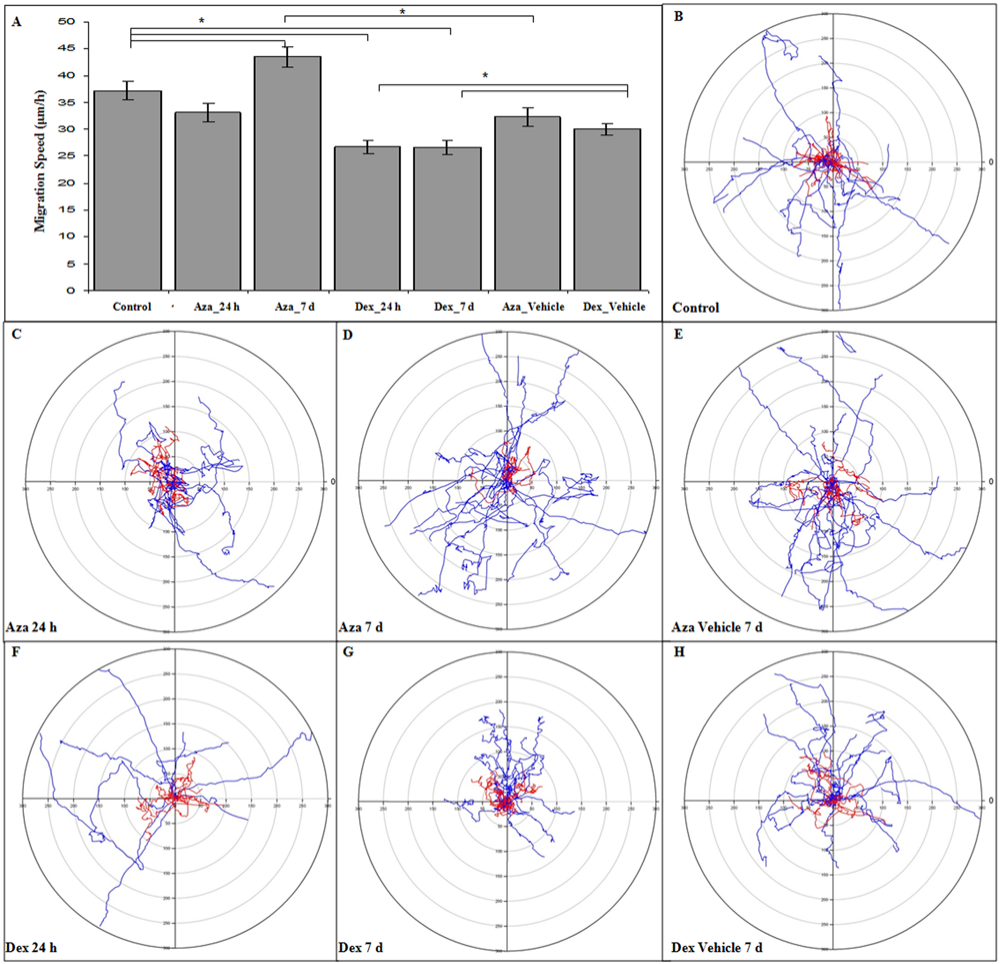
MSC migration speed and spatial trajectory. MSCs were cultured for 24 h or 7 d with or without AZA 1 μM or DEX 10 μM, plated on fibronectin 2 μg/mL and imaged using time-lapse analysis. **(A)** DEX treatment induced a decrease in migration speed, while AZA was associated with a faster motility property. The ST of each migratory cell was accessed through the use of X and Y values, which were normalized to start at a virtual migratory starting point (X = 0 and Y = 0) **(B-H)**. Individual lines on polar plots represent the ST of each cell, while red lines represent cells that stayed close to the starting point and blue lines represent cells that were more exploratory. DEX-treated cells were associated with a lower ST, while AZA showed a tendency to induce a more exploratory phenotype. *P < 0.05, *n* = 4. MSC migration speed was analyzed through ANOVA post-hoc Tukey HSD, and comparisons were made between all groups; the differences among the main groups are represented in the graphic.

The MSC homing capacity towards inflammation and injured sites is an essential process to a successful cell therapy. MSC migration speed and spatial trajectory under 24 h or 7 d of immunosuppressant treatment were assessed through time-lapse analysis. The vehicle treatment had no influence on migration speed when compared to the control group (Fig. 7A). The 24 h AZA treatment had a small effect on cell migration; however, a 24.35% increase was observed after the 7 d treatment in comparison to control group (*P* < 0.05, *n* = 4). DEX treatment impaired the cell speed after 24 h and after 7 d by-28.69% and -25.37%, respectively, in comparison to control group (*P* < 0.05, *n* = 4). The analysis of ST showed that the control group (S4 Movie) presented a high number of migratory cells that were able to reach long distances from the starting migratory point and a few cells that did not explore the field (Fig. 7B). The 7 d vehicle treatment showed no changes in MSC migration when compared to the control group (Fig. 7E, H, S5 and S6 Movies). The AZA 24 h treatment induced no change in cells with a high ST (Fig. 7C, S7 Movie), while the 7 d AZA treatment increased the number of cells that migrated long distances (Fig. 7D, S8 Movie). The DEX 24 h treatment resulted in cells with low ST (Fig. 7F, S9 Movie), and this effect was more pronounced after 7 d of treatment (Fig. 7G, S10 Movie). Taken together, these results suggest that, after 7 d treatment, AZA might improve MSC migration, while DEX would impair MSC migratory capacity.

## Discussion

The currently available IBD treatments rely on immunosuppressive drugs and glucocorticoids but are not entirely effective, and remission often remains difficult to maintain [1,2]. As a novel IBD therapeutic approach, MSCs have emerged as potent regulators of the immune response [7–9], but little is known about their interaction with immunosuppressive drugs. In addition to controversies surrounding MSC homing, it has been previously shown that these cells do interact with endothelial cells through coordinated rolling and adhesion [61]. Furthermore, our group has recently published a study on different routes of MSC administration in experimental UC and showed that the intravenous route can ameliorate the clinical and histological status of the animals [62]. There is evidence of the benefits from local delivery of MSCs in therapy [28,63]; however, intravenous administration of MSCs is a convenient, minimally invasive way that opens options for repeated doses of cell therapy and for cells to reach surgically unavailable sites. Considering that confluence of cells in culture, MSC passage number and the number of cells administered may already modify MSC homing capacity [24,64], it is imperative to better understand how MSCs interact with the immunosuppressive drugs conventionally used for IBD.

We observed herein that the clinical concentrations of the IBD drugs AZA and DEX did not substantially affect the viability of human-derived MSCs. Previous studies have tested different concentrations of DEX and AZA in order to register any changes in cell viability [48,51]. Corroborating with our data, it was postulated that neither drug has an influence on human MSC viability at a variable range of times. However, the glucocorticoid DEX was shown to affect cell viability at high concentrations (up to 1 mg/ml) [48,51]. We used lower drug concentrations in accordance with the drug levels that would be found in the serum of IBD patients under conventional treatment and to which MSCs would be exposed. It is possible that the non-toxic effect of DEX observed in our study could be explained by its action of reducing cell density-related apoptosis in MSCs [65].

In addition to no effects on cell viability, we found that DEX and AZA were able to alter the cell shape and cell migratory behavior. Cell migration depends on the activation of several signaling pathways that result in the break of cell asymmetry, where a more elongated cell correlates to a more effective migratory process [60,66]. Using a polarity index distribution, we demonstrated that 7 days of AZA treatment could indicate a more elongated cell shape, while DEX resulted in a more rounded morphology. The analysis of the cytoskeletal organization, the distribution of FAK and the analysis of membrane dynamics using time-lapse video corroborated with the polarity index because the control and AZA-treated cells showed the presence of stable protrusions, while DEX-rounded shape cells showed a higher presence of ventral stress fibers and unstable protrusions. In this way, Chen et al. (2013) [67] demonstrated that DEX induced the presence of stress fibers on epithelial cells that involved Stomatin expression and F-actin rearrangements. It was shown that endothelial cells treated with DEX presented higher levels of caveolin-1 protein, a regulatory protein of cell surface receptor also involved in the recycling of adhesion receptors [68,69].

It is possible that the immunosuppressant drugs might impair the balance of signaling pathways related to cell migration, such as FAK, which can be associated with activators and/or inhibitors of small GTPases (such as Rac) and modify the polymerization or stabilization of the actin cytoskeleton [70]. For instance, the RhoGTPases are involved in the control of the main migration steps; Rac1 is associated with a fast actin dynamic, while RhoA is associated with adhesion maturation and cell contractility [60]. Our results suggest that the higher presence of dorsal actin fibers associated with small adhesions containing FAK at the cell border indicate that AZA might affect the activity of Rac1, while the association of a higher presence of ventral stress fibers and large adhesions induced by DEX indicate an increase in RhoA activity. However, more experiments are necessary to demonstrate a direct action of immunosuppressive drugs on cell migration signaling.

The differences in cell polarity and actin organization induced by AZA and DEX might reflect a differential effect of immunosuppressant drugs on the MSC migratory ability. Changes in cell polarity and actin organization might reflect alterations in the cell migratory properties [60,66], which are essential for the MSC homing process. We observed that 7 days of AZA treatment resulted in a significant increase in cell speed and spatial trajectory when compared to the control group. Poppe et al. (2006) [71] showed that AZA suppressed T cells’ ability to create flexible regions in the plasma membrane and lamellipodia, concluding that AZA could block Ezrin-Radixin-Moesin (ERM) protein dephosphorylation. Interestingly, in our work, AZA treatment partially preserved these structures on MSCs. It is possible that these changes might be associated with adhesion signaling because leukocytes might be considered non-adherent cells. However, it is still unknown how AZA acts on MSCs because previous studies have mainly focused on explaining the mechanisms affecting immune cells. Additionally, AZA could be positive for stem cell therapy since Mancheño-Corvo et al. (2013) [72] demonstrated an important role of this immunosuppressant as a protective drug towards MSCs due to a reduction in the activation and degranulation of natural killer cells, reducing the cell-mediated lysis of allogeneic MSCs. This study speculates that patients could benefit from concomitant treatment with AZA since the clearance of allogeneic MSCs might be delayed, creating an extended time window for these cells to perform their therapeutic effect. Along with our results regarding increased migratory speed, this immunosuppressant may be able to improve homing of MSCs towards the injury site, enabling a better cell therapy outcome.

Regarding DEX treatment, we found that after 24 h, there was a decrease in cell speed and spatial trajectory, which became more evident after 7 d of treatment. These results might be associated with the higher presence of stress fibers observed following DEX treatment. Similar MSCs shape alterations were described by Geiβler et al. (2012) [43], who observed that chronological and *in vitro* aging promoted round-shaped MSCs and diminished their migration potential, along with a decrease in the expression of genes associated with cytoskeletal organization. Another study demonstrated that DEX treatment induced a more rigid cell structure and impaired T cell migration in a mechanism that involves the activation of the ERM complex, which is related to cytoskeletal rearrangements [73]. In addition to its effects on *in vitro* cell migration, a recently published paper demonstrated that DEX can abolish MSC therapeutic effects *in vivo* in an animal model of liver inflammation by inhibition of MSC nitric oxide synthase, recommending that concomitant treatment with steroids and MSCs should be avoided [74].

Despite the controversies involving migration of MSCs to damaged tissues, the homing process continues to be a key step for MSCs to participate in tissue repair and immunomodulation. Several factors are being identified as regulators of this process and studied in order to enhance cellular migration and improve cell therapy [75–77]. Additionally, studies have explored the interaction between MSC migration and drugs, aiming to better understand this process and improve cell therapy. A recent study tested the multi-tyrosine kinase inhibitor sorafenib on endometrial derived-MSCs and demonstrated that the increased migratory capacity of the cells could be reverted by this drug through the inhibition of ezrin (an ERM component), which was an important finding targeting the eradication of endometriotic implants [78]. Xinaris et al. (2013) [79] demonstrated that MSC preconditioning with insulin-like growth factor-1 (IGF-1) resulted in the improvement of cell migration and the restoration of normal renal function in an animal model, supporting the idea that improving MSC migration could increase therapeutically relevant effects.

Our study aimed to better understand the influence of the conventional treatment of IBD on MSCs, focusing on the main key to a successful therapy. DEX decreased cell motility while AZA ameliorated cellular migration after a prolonged treatment, and both results were influenced by cellular morphology and cytoskeletal organization. Further studies are needed to better understand these processes; however, these findings provide substantial data for the careful evaluation of patients under DEX treatment for ongoing MSC therapy. On the other hand, AZA could be an addition to combined therapy with MSCs, possibly enhancing the results of cell therapy in IBD.

## Supporting Information

S1 Fig Polarity index analysis. Polarity index was calculated as the length of the major migration axis parallel to the direction of movement (a) divided by the length of the perpendicular axis that intersects the center of the cell nucleus (b). (Bar = 100μm)
(TIF)

S2 Fig Cytoskeletal morphology. MSCs were cultured for 24 h, 48 h or 7 d with or without drugs, plated on fibronectin-coated dish overnight, fixed and stained for actin analysis. Results showed that control **(A-C)** and vehicle-treated **(J-O)** cells presented lamellipodia (arrowheads) and a small amount of stress fibers in the cell body. After incubation with AZA for 24 h and 7 d **(D-F)** it was observed the presence of lamellipodia in some cells (arrowheads) and a few membrane projections (arrows). DEX-treated cells showed a decrease on lamellipodia after 24 h **(G)**, which was accompanied by an increase in the presence of thin membrane projections (**H** and **I**, arrows) and a more intense presence of actin stress fibers. (Bar = 100μm); nuclei staining = DAPI.
(TIF)

S1 Movie Control. Movie shows the migratory activity of mesenchymal stem cells cultured with standard media in the presence (right cell) or absence (left cell) of DMSO (0.5%) for 7 days and plated under migration promotion conditions. Total time = 50 min, time interval = 10 s. *n* = 4.
(MOV)

S2 Movie Azathioprine. Movie shows the migratory activity of mesenchymal stem cells cultured with standard media in the presence of AZA (1 μM) for 24 h (left cell) or 7 days (right cell) and plated under migration promotion conditions. Total time = 50 min, time interval = 10 s. *n* = 4.
(MOV)

S3 Movie Dexamethasone. Movie shows the migratory activity of mesenchymal stem cells cultured with standard media in the presence of DEX (10 μM) for 24 h (left cell) or 7 days (right cell) and plated under migration promotion conditions. Total time = 50 min, time interval = 10 s. *n* = 4.
(MOV)

S4 Movie Control. Movie shows the migratory activity of mesenchymal stem cells cultured with standard media and plated under migration promotion conditions. This movie corresponds to Fig. 5B. Total time = 20 h, time interval = 10 min. *n* = 4.
(MOV)

S5 Movie AZA Vehicle 7 d. Movie shows the migratory activity of mesenchymal stem cells cultured with AZA vehicle for 7 d and plated under migration promotion conditions. This movie corresponds to Fig. 5E. Total time = 20 h, time interval = 10 min. *n* = 4.
(MOV)

S6 Movie DEX Vehicle 7 d. Movie shows the migratory activity of mesenchymal stem cells cultured with DEX vehicle for 7 d and plated under migration promotion conditions. This movie corresponds to Fig. 5H. Total time = 20 h, time interval = 10 min. *n* = 4.
(MOV)

S7 Movie Azathioprine 24 h. Movie shows the migratory activity of mesenchymal stem cells cultured with AZA for 24 h and plated under migration promotion conditions. This movie corresponds to Fig. 5C. Total time = 20 h, time interval = 10 min. *n* = 4.
(MOV)

S8 Movie Azathioprine 7 d. Movie shows the migratory activity of mesenchymal stem cells cultured with AZA for 7 d and plated under migration promotion conditions. This movie corresponds to Fig. 5D. Total time = 20 h, time interval = 10 min. *n* = 4.
(MOV)

S9 Movie Dexamethasone 24 h. Movie shows the migratory activity of mesenchymal stem cells cultured with DEX for 24 h and plated under migration promotion conditions. This movie corresponds to Fig. 5F. Total time = 20 h, time interval = 10 min. *n* = 4.
(MOV)

S10 Movie Dexamethasone 7 d. Movie shows the migratory activity of mesenchymal stem cells cultured with DEX for 7 d and plated under migration promotion conditions. This movie corresponds to Fig. 5G. Total time = 20 h, time interval = 10 min. *n* = 4.
(MOV)
