# Supplementary figures and images for: Dexamethasone and Azathioprine Promote Cytoskeletal Changes and Affect Mesenchymal Stem Cell Migratory Behavior
Source: PLoS One. 2015 Mar 10;10(3):e0120538. doi: 10.1371/journal.pone.0120538 (PMC4355407; doi:10.1371/journal.pone.0120538)

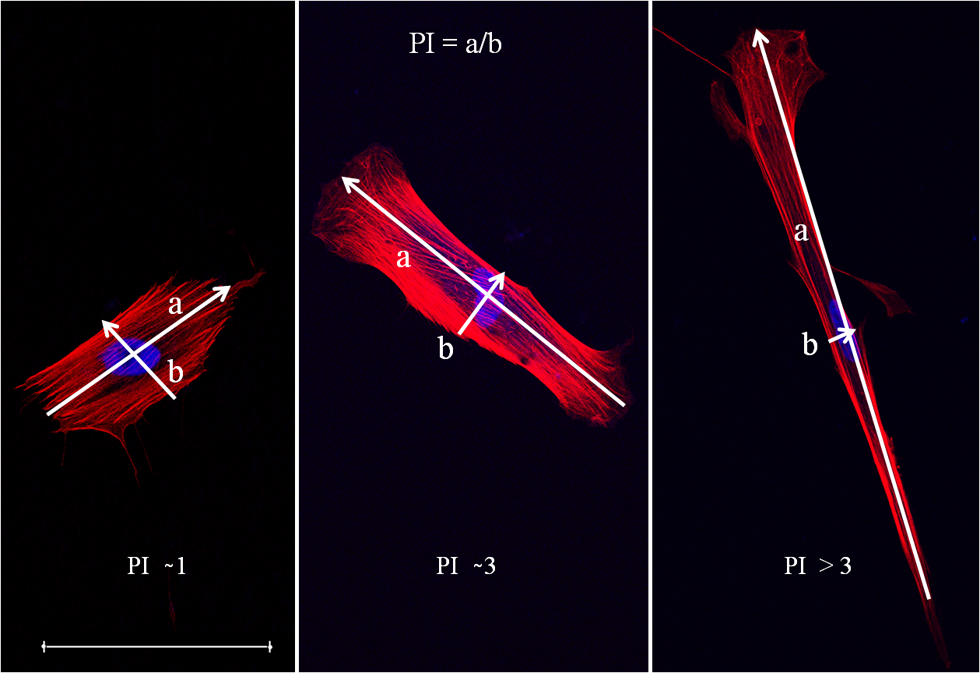

Supplement: S1 Fig — Polarity index was calculated as the length of the major migration axis parallel to the direction of movement (a) divided by the length of the perpendicular axis that intersects the center of the cell nucleus (b). (Bar = 100μm) (TIF) [file pone.0120538.s001.tif]

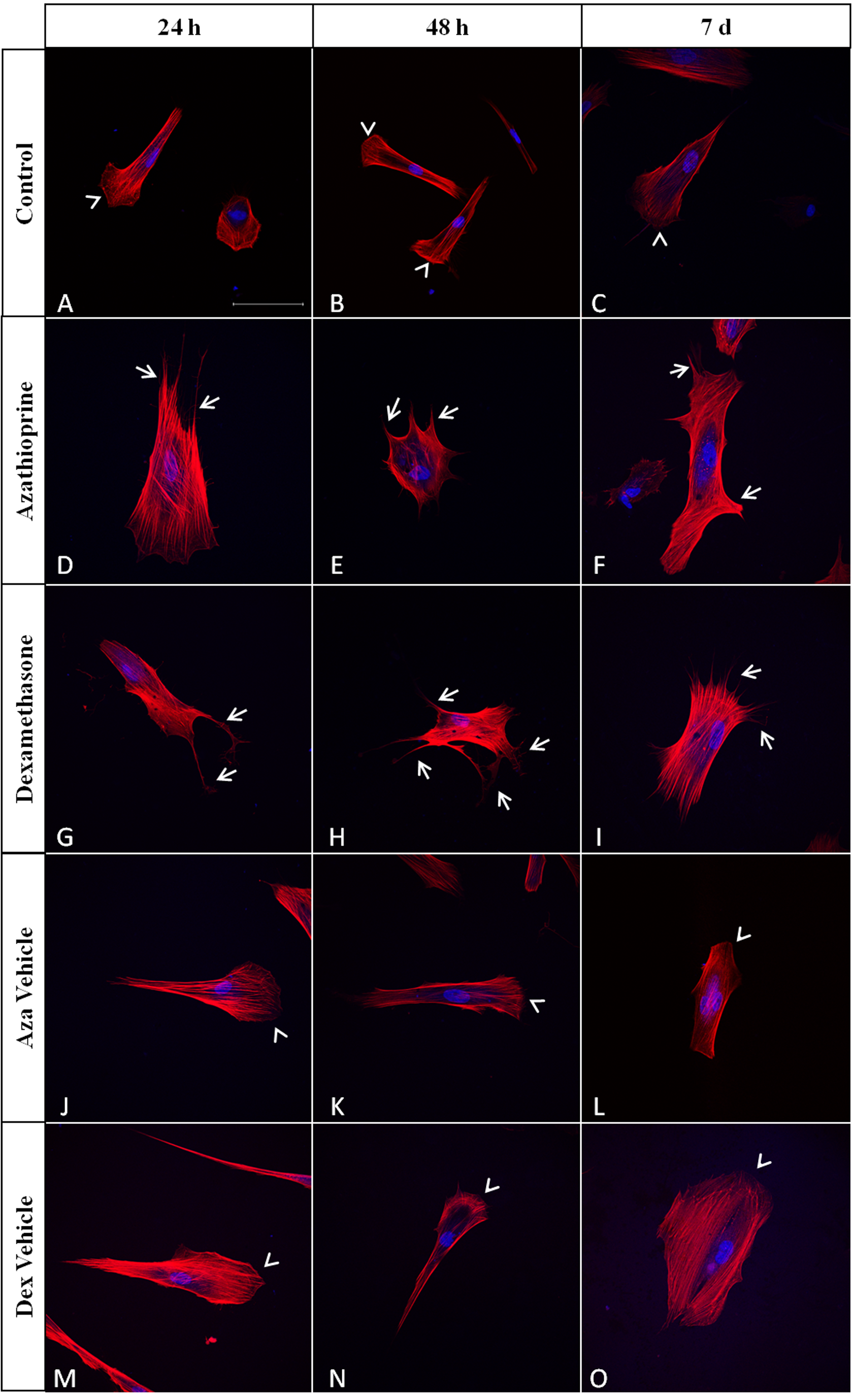

Supplement: S2 Fig — MSCs were cultured for 24 h, 48 h or 7 d with or without drugs, plated on fibronectin-coated dish overnight, fixed and stained for actin analysis. Results showed that control (A-C) and vehicle-treated (J-O) cells presented lamellipodia (arrowheads) and a small amount of stress fibers in the cell body. After incubation with AZA for 24 h and 7 d (D-F) it was observed the presence of lamellipodia in some cells (arrowheads) and a few membrane projections (arrows). DEX-treated cells showed a decrease on lamellipodia after 24 h (G), which was accompanied by an increase in the presence of thin membrane projections (H and I, arrows) and a more intense presence of actin stress fibers. (Bar = 100μm); nuclei staining = DAPI. (TIF) [file pone.0120538.s002.tif]
